# A unique modular implant system enhances load sharing in anterior cervical interbody fusion: a finite element study

**DOI:** 10.1186/1475-925X-13-26

**Published:** 2014-03-11

**Authors:** Vivek Palepu, Ali Kiapour, Vijay K Goel, James M Moran

**Affiliations:** 1 Engineering Center for Orthopaedic Research Excellence (E-CORE), Departments of Bioengineering and Orthopaedic Surgery, Colleges of Engineering and Medicine, University of Toledo, Toledo, OH 43606, USA; 2 RSB Spine LLC, 2530 Superior Ave, Suite 703, Cleveland, OH 44114, USA

## Abstract

**Background:**

The efficacy of dynamic anterior cervical plates is somewhat controversial. Screws in static-plate designs have a smaller diameter and can cut through bone under load. While not ideal, this unintended loosening can help mitigate stress shielding. Stand-alone interbody devices with integral fixation have large endplate contact areas that may inhibit or prevent loosening of the fixation. This study investigates the load sharing ability of a novel dynamic plate design in preventing the stress shielding of the graft material compared to the non-dynamic devices.

**Methods:**

An experimentally validated intact C5-C6 finite element model was modified to simulate discectomy and accommodate implant-graft assembly. Four implant iterations were modeled; InterPlate titanium device with dynamic surface features (springs), InterPlate titanium non-dynamic device, InterPlate titanium design having a fully enclosed graft chamber, and the InterPlate design in unfilled PEEK having a fully enclosed graft chamber. All the models were fixed at the inferior-most surface of C6 and the axial displacement required to completely embed the dynamic surface features was applied to the model.

**Results:**

InterPlate device with dynamic surface features induced higher graft stresses compared to the other design iterations resulting in uniform load sharing. The distribution of these graft stresses were more uniform for the InterPlate dynamic design.

**Conclusions:**

These results indicate that the dynamic design decreases the stress shielding by increasing and more uniformly distributing the graft stress. Fully enclosed graft chambers increase stress shielding. Lower implant material modulus of elasticity does not reduce stress shielding significantly.

## Introduction

Neck pain is one of the most common musculoskeletal conditions and affects 70% of adults at some point in their lives [1]. Substantial disability and economic cost are associated with this pain [2,3]. The pain may arise from any of the spinal structures (discs, facets, ligaments, vertebrae, and muscles), but one of the leading causes is spinal instability resulting from degenerative disc conditions of the cervical spine [4,5]. These types of instabilities are treated with anterior cervical discectomy and fusion (ACDF), which was first reported by Robinson and Smith in 1955 and is now a widely practiced cervical spine surgical technique [6]. Due to high rates of pseudoarthrosis and kyphotic deformity in these procedures, the need for an anterior internal cervical fixation device was recognized. This led to the development of the first anterior cervical plate (ACP) and screw system by Bohler in 1964, followed by the evolution of newer ACP system designs [7].

The purpose of these ACP systems is to maintain alignment after deformity correction, retain graft material, prevent graft collapse and kyphotic deformity, promote arthrodesis, allow early mobilization, and prevent excessive post-operative immobilization. The first generation of ACP devices had unlocked and non-rigid bicortical screws with noted complications such as screw backout and breakage, graft subsidence, and excessive fluoroscopy exposure time. Second-generation devices featured rigid locking unicortical screws that presented new complications such as screw placement challenges, screw-bone interface failure, and graft subsidence with resultant pseudoarthrosis. The introduction of polyaxial screws and partial screw locking mechanisms in the third generation resulted in “windshield wipering” of screws due to screw-bone interface failures [7-12].

A later generation of plates with rotational and translational screws was intended to prevent the screw-bone interface failures [8,13]. However, the efficacy of dynamic anterior cervical plates is controversial.

Interbody devices with integral fixation (anterior cervical implant with screws integrated with the cage) have been developed to overcome some inherent anterior plate design problems, namely their high profile on the anterior surface of the vertebrae and the potential for plate and screw impingement on adjacent levels known to cause disease. Interbody devices with integral fixation typically have a large endplate contact area that may promote stress shielding of graft material to a greater extent than static anterior plates. No provision for dynamic performance is included in most of the currently available interbody device designs.

**Figure 1:**
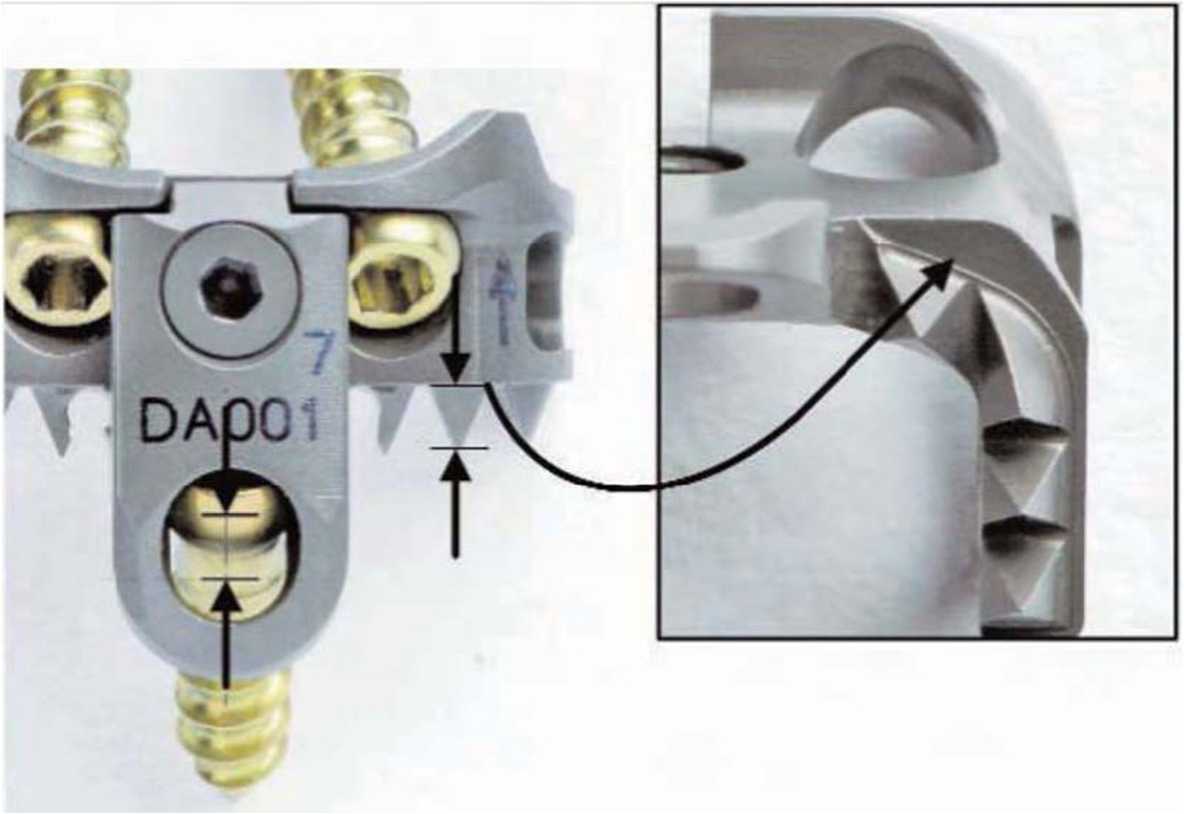
**InterPlate design.** The height of the teeth is matched to the length of screw travel in the slot. When the teeth are fully embedded and the screw has reached the end of the slot, the device rests on flats of the caudal surface (inset).

This study investigates the load sharing ability of a novel dynamic interbody fusion implant design (Figure 1), the InterPlate®, intended to prevent stress shielding of the graft by providing prominent surface features to penetrate vertebral bone and screws with sufficient degrees of freedom to permit this penetration to occur.

## Methods

**Figure 2:**
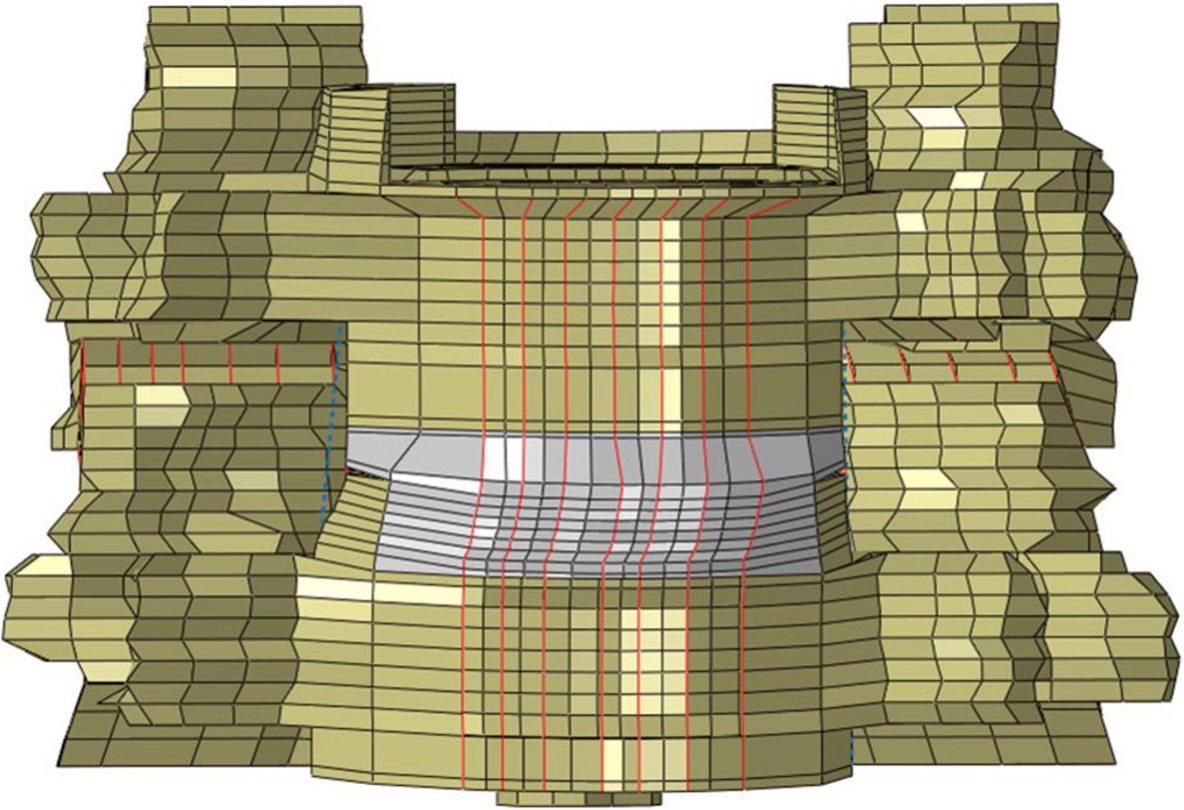
Anterior view of the experimentally validated osseo-ligamentous C5-C6 FSU finite element model.

An intact C5-C6 ligamentous cervical functional spinal unit (FSU) model comprising 5,577 elements and 4,219 nodes was used for this study (Figure 2). The geometric data of the C5-C6 FSU was obtained from the computed tomography scans (transverse slices 1 mm thick) of a cadaveric ligamentous spine specimen. Sequentially stacked, digitized cross-sectional data provided the means to generate this model. The commercial software Abaqus/Standard™ version 6.11 (Simulia, Inc. Rhode Island, U.S.A.) was used for analysis. This intact spine model has been experimentally validated in earlier studies [14].

The vertebral bodies were modeled as a cancellous bone core surrounded by a 0.5 mm thick cortical shell using three-dimensional (3-D) hexagonal elements (C3D8). The posterior bone regions were constructed of C3D8 elements, all of which were assigned a single set of material properties, as shown in the following table (Table 1). The facet joints were simulated with 3-D gap contact elements. These elements transferred force between nodes along a single direction as a specified gap between these nodes closed. The cartilaginous layer between the facet surfaces was simulated by Abaqus’ “softened contact” parameter, which exponentially adjusted force transfer across the joint depending on the size of the gap. An initial gap of 0.5 mm, as found for actual cadaveric specimens, was specified. At full closure, the joint assumed the same stiffness as the surrounding bone.

**Table 1:** Material properties of elements used in the model

**Element group name**	**Element type**	**Young’s Modulus (MPa)**	**Poisson's ratio**
**Cortical bone**	Isotropic, elastic hex elements **(C3D8)**	**10,000**	**0.3**
**Cancellous bone**	Isotropic, elastic hex elements **(C3D8)**	**450**	**0.25**
**Posterior bone**	Isotropic, elastic hex elements **(C3D8)**	**3500**	**0.25**
**Annulus ground substance**	Isotropic, elastic hex elements **(C3D8)**	**4.2**	**0.25**
**Annulus fibers**	REBAR elements	**-**	**0.45**
**Nucleus pulposus**	Incompressible fluid, cavity elements	**1**	**0.49**
**Anterior Longitudinal Ligament (ALL)**	Tension-only, Truss elements **(T3D2)**	15 (<12%*) 30 (>12%*)	**0.3**
**Posterior Longitudinal Ligament (PLL)**	Tension-only, Truss elements **(T3D2)**	10 (<12%*) 20 (>12%*)	**0.3**
**Ligamentum Flavum (LF)**	Tension-only, Truss elements **(T3D2)**	7 (<12%*) 30 (>12%*)	**0.3**
**Interspinous Ligament (ISL)**	Tension-only, Truss elements **(T3D2)**	5(<25%*) 10 (>25%*)	**0.3**
**Capsular Ligaments (CAP)**	Tension-only, Truss elements **(T3D2)**	15 (20-40%*) 30 (>40%*)	**0.3**
**T itanium (InterPlate)**	Isotropic, elastic Tetrahedral elements **(C3D4)**	**1,130,000**	**0.34**
**Cortical Bone Graft**	Isotropic, elastic Tetrahedral elements **(C3D4)**	**10,000**	**0.3**
**PEEK (InterPlate)**	Isotropic, elastic Tetrahedral elements **(C3D4)**	**3400**	**0.4**

*Strain Values.

The fissure of Luschka’s joint was modeled similarly using gap elements. When the gap across the fissure was closed, all resulting deformation came from compression of the elements of the annulus fibrosus. The intervertebral disc was modeled as a composite of a solid matrix with embedded fibers, via the REBAR parameter, in concentric rings around a pseudo-fluid nucleus. Seven concentric rings of ground substance each contained two evenly spaced layers of fibers (plus one ground substance ring with one layer of fibers) oriented at ± 65° to the vertical axis. Fiber thickness and stiffness increased in the radial direction. Implementing the “no compression” option restricted the annulus fibers to resisting tension only.

The nucleus pulposus was modeled as an incompressible fluid with a very low stiffness (1 MPa) and near incompressibility (i.e., Poisson’s ratio of 0.4999). All seven major spinal ligaments were represented and assigned nonlinear material properties. Nonlinear ligament stiffness (low stiffness at low strains followed by increasing stiffness at higher strains) was simulated through the “hypoelastic” material designation, which allowed the definition of the axial stiffness as a function of axial strain. Three dimensional 2-noded truss elements were used to construct the ligament.

**Figure 3:**
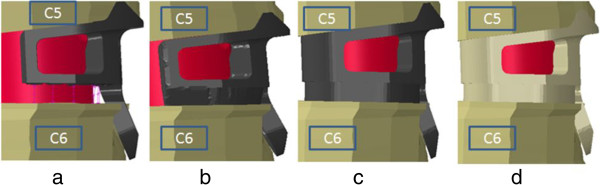
**Enlarged view of finite element models of the four implant iterations with cortical bone graft (pink). (a)** InterPlate titanium device with dynamic surface features (springs), **(b)** InterPlate titanium non-dynamic device without teeth, **(c)** InterPlate design in titanium without teeth and having a fully enclosed graft chamber, **(d)** InterPlate design in unfilled PEEK without teeth and having a fully enclosed graft chamber.

The intact model was modified to simulate discectomy and accommodate the implant-cortical bone graft assembly. Four iterations (two InterPlate iterations and two additional models representing common interbody device with integral fixation design concepts) were modeled (Figure 3):

• The InterPlate titanium design, as commercially available (dynamic).

• The InterPlate design without teeth representing a non-dynamic device.

• The InterPlate design in titanium without teeth and having a fully enclosed graft chamber.

• The InterPlate design in unfilled PEEK without teeth and having a fully enclosed graft chamber.

The titanium (Elastic modulus of 113 GPa; Poisson’s ratio of 0.34) and PEEK (Elastic modulus of 3.5 GPa; Poisson’s ratio of 0.4) material properties were assigned to the respective implants. Bone compaction caused by teeth penetrating bone was modeled as follows. For the dynamic titanium device, the surface features were replaced with springs in order to accurately represent tooth penetration and screw sliding. The spring constants reproduce the load-deflection curve for the InterPlate alone, as determined by ASTM F2267 - Test of Load Induced Subsidence [15].

The top and bottom surfaces of the cortical bone graft were tied to the respective top and bottom endplates of the vertebrae. Sliding contact was simulated using the contact pair option in Abaqus between the InterPlate and the anterior portion of the C5-C6 motion segment. All models were fixed at the inferior-most surface of C6 and the displacement required to completely embed the surface features of the plate was applied to the C5 superior surface of the model. Three-dimensional plots of graft stresses were generated for each iteration using scientific visualization software (Visual Data, GraphNow, Issaquah, WA).

## Results

**Figure 4:**
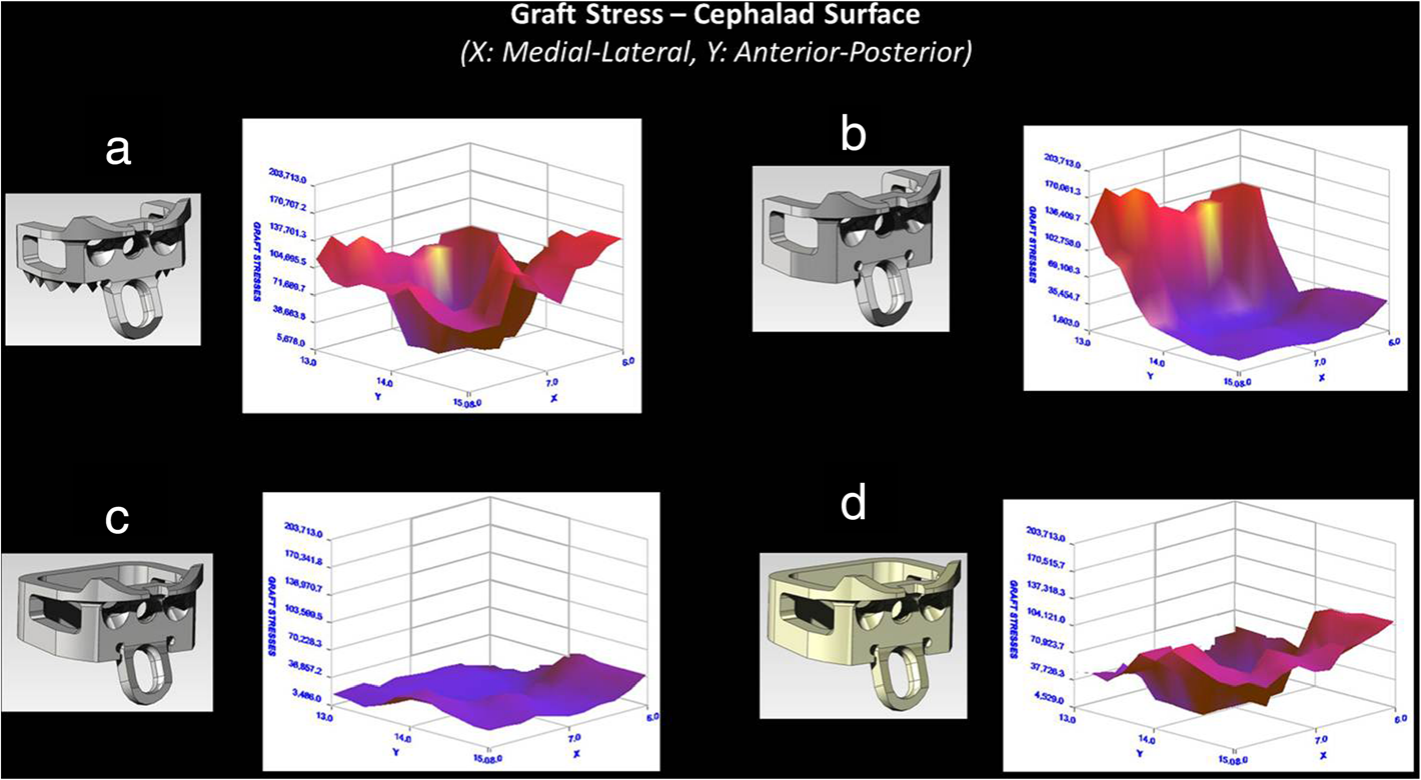
**Three-dimensional plots of stress on cephalad graft surface for different iterations of the InterPlate design. (a)** The InterPlate titanium device having dynamic surface features, **(b)** The InterPlate design without teeth representing a non-dynamic device. The plate shields the anterior portion of the graft, **(c)** The InterPlate design in titanium without teeth and having a fully enclosed graft chamber, **(d)** The InterPlate design in unfilled PEEK without teeth and having a fully enclosed graft chamber.

Graft stresses were higher and more symmetrically distributed for the InterPlate titanium device having dynamic surface features than for the other modeled implants (Figure 4a). Maximum stress in the graft with this dynamic device was 1.95 GPa. When the surface features were removed, the metal implant stress shielded the anterior half of the graft (Figure 4b), reducing graft stress in that location by approximately 75%. The unshielded posterior graft was subjected to higher stress with a maximum value of 2.08 GPa.

Other cases with fully enclosed graft chambers (Both Titanium and PEEK implants) significantly stress shielded the graft material contained within. The titanium implant with an enclosed graft chamber uniformly decreased the graft stress by about 75% (Figure 4c). The PEEK device with an enclosed graft chamber decreased graft stress by approximately 75% posteriorly and 25% anteriorly (Figure 4d). Maximum stress values in the graft were 0.48 GPa for titanium implant with an enclosed graft chamber and 1.26 GPa for PEEK device with an enclosed graft chamber respectively.

## Discussion

Finite element and animal studies of cage designs indicate that excessive stress shielding can inhibit fusion [16,17]. However, some degree of stabilization is required for fusion to occur reliably. Somewhere between unrestrained motion and infinitely rigid fixation a range of acceptable or optimal load sharing must exist.

Some anterior plates address stress shielding by incorporating dynamic load-sharing design features. A biomechanical study comparing load sharing of static and dynamic plate configurations was conducted using a C4-C7 finite element model [18]. The study demonstrated that a locking plate carried the majority of the load (>90%) in all simulations and the dynamic plate shared a greater portion of load through the cage (up to 40%). A study by Ghahreman et al. showed that dynamic plates provide fusion rates and clinical results comparable to ACDF static Plates [19].

The in vitro studies comparing static and dynamic plates are controversial because of the fusion rates observed clinically for static plates. However, a study by Han et al. found that static plates promote clinical fusions by dynamizing due to screw migration through the vertebral bodies [20]. In addition to screw migration and loosening, static plates also can behave as dynamic plates via screw or plate fracture. Other more general complications associated with anterior plates are screw intrusion into adjacent disc spaces and excess plate length, both of which have been implicated in adjacent level deterioration [21]. These issues have led to another generation of implants, interbody fixation devices with integral fixation.

Studies have shown interbody devices with integral fixation can provide stabilization comparable to anterior devices. Scholz et al. suggested that the integrated plate-spacer provided comparable stability to traditional spacer and plate constructs while preventing several aspects of perioperative and postoperative morbidity [5]. Other studies were also in agreement that the integrated plate-spacer system provided adequate biomechanical stability compared to traditional methods and may potentially reduce perioperative and postoperative complications [22,23].

**Figure 5:**
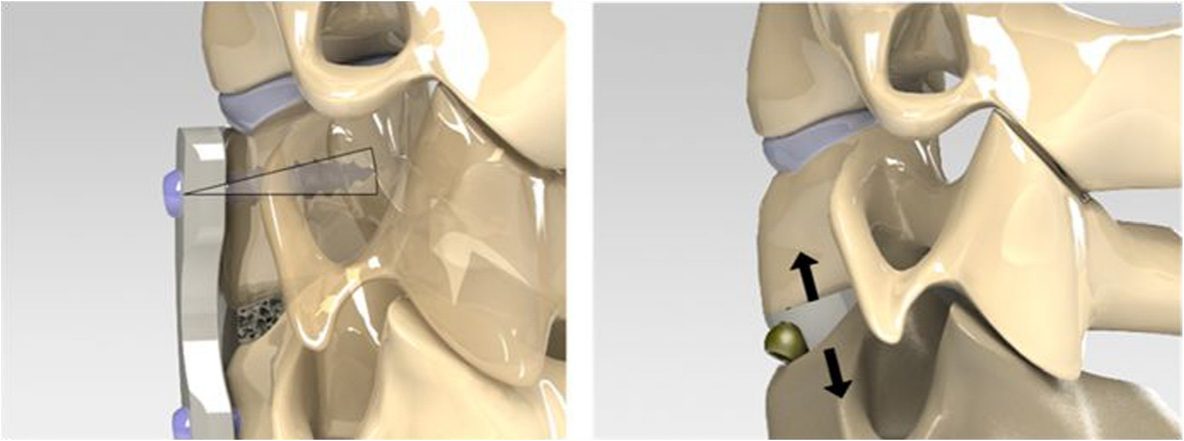
**Static anterior plates can dynamize as a result of screw loosening or fracture (left).** Interposing a rigid (static) device between the vertebrae may prohibit this method of dynamization, resulting in stress shielding of the graft (right).

However, because the interbody devices with integral fixation are located within the disc space and have relatively large contact areas on the endplate, they may not permit dynamization by screw migration through cancellous bone as occurs with anterior plates (Figure 5).

Even screws with rotational degrees of freedom may essentially be locked if the interbody device inhibits vertebral movement. This suggests the potential need for an integrated plate-spacer system that has dynamic features to enable load sharing with the graft in addition to the inherent advantages over anterior plates.

As noted previously the InterPlate is an interbody device with integral fixation that accommodates screw rotation and translation with backout prevention. Like an anterior plate system, the fixation component and graft are not attached. In order to provide a direct comparison in this study, the same shape made of titanium without dynamic performance and similar shapes with enclosed graft chambers constructed of titanium and PEEK were also analyzed.

The fixation means employed by current static interbody designs could be fins, staples, or two to four screws and, except for the InterPlate, the screws are either locked or rotational. To simplify the analyses, it was assumed that whichever fixation means the static designs incorporated did not inhibit load transfer. Screws or staples were not modeled. Locked screws or limited fixation degrees of freedom (e.g., rotation only) will further inhibit load transfer. The experimental ASTM F 2267 test data used to model InterPlate stiffness includes screw fixation, so differences between the dynamic InterPlate case and hypothetical static cases likely would be magnified.

However, FE model used in this study has some limitations. First, the endplate in our model is uniform, with a thickness of 0.5mm, whereas, in reality, the endplate thickness varies from the center to the periphery. However, the variation in thickness is very small and hence would not affect the outcome of our study. Secondly, our model simulates single geometry of the spine model and thus does not account for variations in the patients/cadavers.

It has been shown that interbody devices with integral fixation provide adequate biomechanical stability compared to conventional systems under quasi-static loading [23]. Results from this FE study indicate that this unique device design (interbody device with integral fixation having dynamic features) may enhance load sharing ability and prevent stress shielding compared to other static systems. However, there is paucity in the literature on fatigue implications of these devices. Subsidence, screw breakage and loosening can be some of the major issues associated with these devices when subjected to the fatigue loading. Further research in this direction can help to better understand the device efficacy.

## Conclusions

The results indicated that graft stress is more uniformly distributed for the dynamic InterPlate design. Fully enclosed graft chambers increase stress shielding, and lower implant material modulus of elasticity does little to reduce stress shielding. The most effective way to increase load sharing in interbody devices with integral fixation is to design-in some dynamic mechanism. None of these observations are counterintuitive.

While it is difficult to predict the implications of these observations on clinical performance, this finite element study indicates that the InterPlate dynamic design may reduce the graft stress shielding and thus provide more favorable conditions for successful fusion without graft failure.

## Competing interests

The authors declare that they have no competing interests.

## Authors’ contributions

VP carried out the finite element study, analyses of the results and drafted the manuscript. AK participated in the finite element analysis. VG mentored the finite element analysis and provided valuable suggestions in drafting the manuscript. He was overall responsible for the project and the manuscript. JM provided device files required to carry out the finite element analysis and helped in drafting the manuscript. All authors read and approved the final manuscript.
